# Beta-tubulin and Actin gene phylogeny supports *Phaeoacremoniumovale* as a new species from freshwater habitats in China

**DOI:** 10.3897/mycokeys.41.27536

**Published:** 2018-10-11

**Authors:** Shi-Ke Huang, Rajesh Jeewon, Kevin D. Hyde, D. Jayarama Bhat, Putarak Chomnunti, Ting-Chi Wen

**Affiliations:** 1 Engineering and Research Center of Southwest Bio-Pharmaceutical Resources, Ministry of Education, Guizhou University, Guiyang 550025, China; 2 Center of Excellence in Fungal Research, Mae Fah Luang University, Chiang Rai 57100, Thailand; 3 Key Laboratory for Plant Biodiversity and Biogeography of East Asia (KLPB), Kunming Institute of Botany, Chinese Academy of Sciences, Kunming, 650201, Yunnan, China; 4 Department of Health Sciences, Faculty of Science, University of Mauritius, Reduit, Mauritius; 5 Azad Housing Society, No. 128/1-J, Curca, P.O. Goa Velha 403108, India; 6 Formerly, Department of Botany, Goa University, Goa, 403206, India; 7 School of Science, Mae Fah Luang University, Chiang Rai 57100, Thailand

**Keywords:** 1 new species, Togniniales, Sordariomycetes, Morphology, Phylogeny

## Abstract

A new species of *Phaeoacremonium*, *P.ovale* (Togniniaceae), was isolated during a diversity study of freshwater fungi from Yunnan Province in China. Morphological and cultural studies of the fungus were carried out and its sexual and asexual morphs (holomorph) are introduced herein. This species is characterised by peculiar long-necked, semi-immersed ascomata with oval to ellipsoid ascospores and ellipsoid to ovoid conidia. Phylogenetic analyses of a combined TUB and ACT gene dataset revealed that strains of *P.ovale* constitute a strongly supported independent lineage and are related to *P.griseo-olivaceum* and *P.africanum*. The number of nucleotide differences, across the genes analysed, also supports establishment of *P.ovale* as a new species.

## Introduction

Lignicolous freshwater fungi are important in nutrient recycling (Hyde et al. 2016). A number of taxonomic studies have focused on the diversity of such fungi in the South East Asian region and these investigations have reported a number of novel species (e.g. Jeewon et al. 2003; Cabanela et al. 2007; Zhang et al. 2008; Luo et al. 2018). In this study, we report a new species of *Phaeoacremonium* isolated from decaying wood from a stream in Yunnan Province, China.

*Phaeoacremonium* (= *Togninia*), introduced by Crous et al. (1996), is typified by *P.parasiticum* and it belongs to Togniniaceae (Gramaje et al. 2015). *Phaeoacremonium* was reported to be the asexual morph of *Togninia* (Mostert et al. 2003, 2006a; Pascoe et al. 2004). Gramaje et al. (2015) proposed *Phaeoacremonium* over *Togninia* as the correct name based on common usage and this has been listed in Réblová et al. (2016) and followed in Wijayawardene et al. (2018). The species are basically characterised by black ascomata with a long neck and clavate to cylindrical asci with oval to ellipsoid, hyaline ascospores and straight or flexuous mononematous conidiophores with oval to reniform phialo-conidia (Marin-Felix et al. 2018; Spies et al. 2018).

Most species of *Phaeoacremonium* are plant or/and human pathogens and some have been recorded on arthropods or in soil (Groenewald et al. 2001; Guarro et al. 2003; Hemashettar et al. 2006; Mostert et al. 2006a; Damm et al. 2008; Gramaje et al. 2015) while others are causal agents of Petri disease and esca of grapevines (Pascoe et al. 2004; Rooney-Latham et al. 2005a; Mostert et al. 2006b). *Phaeoacremonium* species can also infect a wide range of woody hosts, such as cherry, apricot, olive and peach trees (Rumbos 1986; Di Marco et al. 2004; Kubátová et al. 2004). Recent studies have reported the importance of *Phaeoacremonium* species in causing brown wood streaking of *Olea* spp. and *Prunus* spp. (Mostert et al. 2006b; Damm et al. 2008; Gramaje et al. 2012; Nigro et al. 2013; Olmo et al. 2014; Carlucci et al. 2015). Rooney-Latham et al. (2004, 2005a, b) reported that, in the presence of water, spores in some *Phaeoacremonium* species are forcibly discharged from perithecia through the long neck and exit the ostiole to be dispersed by wind, rain or insects in order to colonise other substrates. Recently Hu et al. (2012) introduced a freshwater inhabiting species, *Phaeoacremoniumaquaticum* (= *Togniniaaquatica*).

Species of Togniniaceae have been reported to colonise substrates in different types of habitats and recent taxonomic studies have revealed additional new species (Gramaje et al. 2015). We have been studying fungi along a north-south gradient in the Asian region (Hyde et al. 2016) and, in this study, we report on two collections of *Phaeoacremonium* from China. The aim here is to characterise these two strains as one novel species based on morphology as well as to investigate their phylogenetic affinities with previously known Togniniaceae species based on partial TUB and ACT genes.

## Materials and methods

### Sample collection, morphological studies and isolation

Submerged dead wood was collected from Baoshan, Yunnan Province in China in October 2016, brought to the laboratory in zip lock plastic bags and treated in the laboratory following procedures detailed in Luo et al. (2018). Fruiting bodies were found growing on decaying wood in a sterile plastic box after two weeks of incubation and the fungus was subsequently isolated based on the method of Chomnunti et al. (2014). Specimens were examined by a Motic SMZ 168 stereomicroscope. Micromorphological characters were examined using a Nikon ECLIPSE 80i compound microscope and images were captured with a Canon EOS 600D digital camera. Identification of colours was based on Ridgway (1912). The Taro soft Image Framework programme version 0.9.0.7 was used for measurements. Single spores were isolated and grown on water agar (WA) and potato dextrose agar (PDA) media. Ascospores germinated on PDA within 1 week. The colonies were transferred to WA and PDA to promote sporulation (sporulation occurred after 30 days in PDA). The cultures were checked 2 to 3 times per week and all procedures were performed in a sterile environment and at room temperature. The morphological characters of the asexual morph were examined after sporulation. Specimens are deposited in the Kunming Institute of Botany, Academia Sinica (KUN) and duplicated in Mae Fah Luang University (MFLU) Herbarium, Chiang Rai, Thailand. Facesoffungi numbers (FoF) (http://www.facesoffungi.org/) were obtained as stated in Jayasiri et al. (2015) and Index Fungorum numbers (IF) (http://www.indexfungorum.org/names/IndexFungorumRegisterName.asp).

### DNA extraction, PCR amplification and sequencing

Total genomic DNA was extracted from mycelium using a Trelief Plant Genomic DNA Kit following the instructions of the manufacturer. The genomic DNA was amplified by using polymerase chain reaction (PCR) in a 25 μl reaction mixture. Partial regions of the beta-tubulin (TUB) and Actin (ACT) gene were amplified using the primer pairs T1 (O’Donnell and Cigelnik 1997) and Bt2b (Glass and Donaldson 1995), ACT-513F and ACT-783R (Carbone and Kohn 1999), respectively. The internal transcribed spacers (ITS) regions of the rDNA (ITS1-5.8S-ITS2) were also amplified using primer pairs ITS5 and ITS4 (White et al. 1990) but no further analyses were done on these due to lack of sequence data. The PCR conditions for these regions were as follows: an initial denaturation at 94 °C for 3 min, followed by 35 cycles of denaturation at 94 °C for 30 sec, annealing at 51 °C (TUB) or 60 °C (ACT) or 55 °C (ITS) for 50 sec and extension at 72 °C for 1 min, with a final extension at 72 °C for 10 min. PCR products were then sequenced with the primers mentioned above by a commercial sequencing provider (Tsingke Company, Beijing, P.R. China).

### Phylogenetic analysis

The quality of the amplified nucleotide sequences was checked and combined by SeqMan version 7.1.0 (44.1) and Finch TV version 1.4.0 (www.geospiza.com). Sequences used by Marin-Felix et al. (2018), Spies et al. (2018) and the closest matches for our strains were retrieved from the National Center for Biotechnology Information (NCBI) by nucleotide BLAST. Sequences were aligned in MAFFT v. 7.310 (http://mafft.cbrc.jp/alignment/server/index.html) (Katoh and Standley 2016) and manually corrected in Bioedit 7.0.9.0 (Hall 1999).

The phylogenetic analyses of combined gene regions (TUB and ACT) were performed using maximum-likelihood (ML) and Bayesian Inference (BI) methods. The best-fit model (GTR+G+I) was obtained using jModelTest 2.1.10 under the Akaike Information Criterion (AIC) calculations (Darriba et al. 2012). The ML analysis was enforced with RAxML-HPC v.8 on XSEDE (Stamatakis 2014; Miller et al. 2015) with 1000 rapid bootstrap replicates. Bayesian inference was implemented by MrBayes v. 3.0b4 (Ronquist and Huelsenbeck 2003). Four simultaneous Markov chains were run for 5,000,000 generations sampling one tree every 1000^th^ generations and other criteria as outlined by Hongsanan et al. (2017). The temperature value was lowered to 0.15, burn-in was set to 0.25. Gaps were treated as missing data with no differential weighting of transitions against transversions and the partition homogeneity test was performed to assess whether datasets from different genes were congruent. Phylogenetic trees were viewed with FigTree v1.4.0 (http:// tree.bio.ed.ac.uk/software/figtree/) and processed by Adobe Illustrator CS5. Alignment and trees were deposited in TreeBASE (submission ID: 22810). The nucleotide sequence data of the new taxon have been deposited in GenBank (Table 1).

**Table 1.** Strains and GenBank accession numbers of the isolates used in this study. Isolates from this study are marked with asterisk (*) and the type strains are in bold.

Species	Voucher/Culture	GenBank accession number	
		TUB	ACT
*** Phaeoacremonium africanum ***	**CBS 120863**	**EU128100**	**EU128142**
*** Phaeoacremonium album ***	**CBS 142688**	**KY906885**	**KY906884**
*** Phaeoacremonium alvesii ***	**CBS 110034**	**AY579301**	**AY579234**
* Phaeoacremonium alvesii *	CBS 729.97	AY579302	AY579235
*** Phaeoacremonium amstelodamense ***	**CBS 110627**	**AY579295**	**AY579228**
*** Phaeoacremonium amygdalinum ***	**CBS 128570**	**JN191307**	**JN191303**
* Phaeoacremonium amygdalinum *	CBS H-20507	JN191305	JN191301
* Phaeoacremonium amygdalinum *	CBS H-20508	JN191306	JN191302
*** Phaeoacremonium angustius ***	**CBS 114992**	**DQ173104**	**DQ173127**
* Phaeoacremonium angustius *	CBS 114991	DQ173103	DQ173126
*** Phaeoacremonium argentinense ***	**CBS 777.83**	**DQ173108**	**DQ173135**
*** Phaeoacremonium armeniacum ***	**ICMP 17421**	**EU596526**	**EU595463**
*** Phaeoacremonium aureum ***	**CBS 142691**	**KY906657**	**KY906656**
*** Phaeoacremonium australiense ***	**CBS 113589**	**AY579296**	**AY579229**
* Phaeoacremonium australiense *	CBS 113592	AY579297	AY579230
*** Phaeoacremonium austroafricanum ***	**CBS 112949**	**DQ173099**	**DQ173122**
* Phaeoacremonium austroafricanum *	CBS 114994	DQ173102	DQ173125
* Phaeoacremonium austroafricanum *	CBS 114993	DQ173101	DQ173124
*** Phaeoacremonium bibendum ***	**CBS 142694**	**KY906759**	**KY906758**
***Phaeoacremoniumcanadens*** e	**PARC327**	**KF764651**	**KF764499**
*** Phaeoacremonium cf. mortoniae ***	**ICMP 18088**	**HM116767**	**HM116773**
*** Phaeoacremonium cinereum ***	**CBS 123909**	**FJ517161**	**FJ517153**
* Phaeoacremonium cinereum *	CBS H-20215	FJ517160	FJ517152
* Phaeoacremonium cinereum *	CBS H-20213	FJ517158	FJ517150
*** Phaeoacremonium croatiense ***	**CBS 123037**	**EU863482**	**EU863514**
*** Phaeoacremonium fraxinopennsylvanicum ***	**CBS 101585**	**AF246809**	**DQ173137**
* Phaeoacremonium fraxinopennsylvanicum *	CBS 110212	DQ173109	DQ173136
*** Phaeoacremonium fuscum ***	**CBS 120856**	**EU128098**	**EU128141**
*** Phaeoacremonium gamsii ***	**CBS 142712**	**KY906741**	**KY906740**
*** Phaeoacremonium geminum ***	**CBS 142713**	**KY906649**	**KY906648**
*** Phaeoacremonium globosum ***	**ICMP 16988**	**EU596525**	**EU595466**
* Phaeoacremonium globosum *	ICMP 17038	EU596521	EU595465
* Phaeoacremonium globosum *	ICMP 16987	EU596527	EU595459
*** Phaeoacremonium griseo-olivaceum ***	**CBS 120857**	**EU128097**	**EU128139**
*** Phaeoacremonium griseorubrum ***	**CBS 111657**	**AY579294**	**AY579227**
* Phaeoacremonium griseorubrum *	CBS 566.97	AF246801	AY579226
*** Phaeoacremonium hispanicum ***	**CBS 123910**	**FJ517164**	**FJ517156**
*** Phaeoacremonium hungaricum ***	**CBS 123036**	**EU863483**	**EU863515**
*** Phaeoacremonium inflatipes ***	**CBS 391.71**	**AF246805**	**AY579259**
* Phaeoacremonium inflatipes *	CBS 113273	AY579323	AY579260
*** Phaeoacremonium iranianum ***	**CBS 101357**	**DQ173097**	**DQ173120**
* Phaeoacremonium iranianum *	CBS 117114	DQ173098	DQ173121
*** Phaeoacremonium italicum ***	**CBS 137763**	**KJ534074**	**KJ534046**
* Phaeoacremonium italicum *	CBS 137764	KJ534075	KJ534047
* Phaeoacremonium italicum *	CBS H-21638	KJ534076	KJ534048
*** Phaeoacremonium junior ***	**CBS 142697**	**KY906709**	**KY906708**
* Phaeoacremonium krajdenii *	CBS 110118	AY579324	AY579261
*** Phaeoacremonium krajdenii ***	**CBS 109479**	**AY579330**	**AY579267**
*** Phaeoacremonium longicollarum ***	**CBS 142699**	**KY906689**	**KY906688**
*** Phaeoacremonium luteum ***	**CBS 137497**	**KF823800**	**KF835406**
*** Phaeoacremonium meliae ***	**CBS 142710**	**KY906825**	**KY906824**
*** Phaeoacremonium minimum ***	**CBS 246.91**	**AF246811**	**AY735497**
* Phaeoacremonium minimum *	CBS 100397	AF246806	AY735498
* Phaeoacremonium mortoniae *	CBS 211.97	AF246810	
*** Phaeoacremonium nordesticola ***	**CMM4312**	**KY030807**	**KY030803**
*** Phaeoacremonium novae-zealandiae ***	**CBS 110156**	**DQ173110**	**DQ173139**
* Phaeoacremonium novae-zealandiae *	CBS 110157	DQ173111	DQ173140
*** Phaeoacremonium occidentale ***	**ICMP 17037**	**EU596524**	**EU595460**
*** Phaeoacremonium oleae ***	**CBS 142704**	**KY906937**	**KY906936**
*Phaeoacremoniumovale	**KUMCC 17-0145**	**MH395327**	**MH395325**
*Phaeoacremoniumovale	**KUMCC 18-001**8	**MH395328**	**MH395326**
*** Phaeoacremonium pallidum ***	**CBS 120862**	**EU128103**	**EU128144**
*** Phaeoacremonium parasiticum ***	**CBS 860.73**	**AF246803**	**AY579253**
* Phaeoacremonium parasiticum *	CBS 113585	AY579307	AY579241
* Phaeoacremonium parasiticum *	CBS 514.82	AY579306	AY579240
*** Phaeoacremonium paululum ***	**CBS 142705**	**KY906881**	**KY906880**
* Phaeoacremonium pravum *	CBS 142686	KY084246	KY084248
*** Phaeoacremonium proliferatum ***	**CBS 142706**	**KY906903**	**KY906902**
*** Phaeoacremonium prunicola ***	**CBS 120858**	**EU128095**	**EU128137**
* Phaeoacremonium prunicola *	CBS 120858	EU128096	EU128138
*** Phaeoacremonium pseudopanacis ***	**CPC 28694**	**KY173609**	**KY173569**
*** Phaeoacremonium roseum ***	**PARC273**	**KF764658**	**KF764506**
*** Phaeoacremonium rosicola ***	**CBS 142708**	**KY906831**	**KY906830**
*** Phaeoacremonium rubrigenum ***	**CBS 498.94**	**AF246802**	**AY579238**
* Phaeoacremonium rubrigenum *	CBS 112046	AY579305	AY579239
*** Phaeoacremonium santali ***	**CBS 137498**	**KF823797**	**KF835403**
*** Phaeoacremonium scolyti ***	**CBS 113597**	**AF246800**	**AY579224**
* Phaeoacremonium scolyti *	CBS 113593	AY579293	AY579225
* Phaeoacremonium scolyti *	CBS 112585	AY579292	AY579223
*** Phaeoacremonium sicilianum ***	**CBS 123034**	**EU863488**	**EU863520**
* Phaeoacremonium sicilianum *	CBS 123035	EU863489	EU863521
*Phaeoacremonium* sp.	KMU 8592	AB986584	AB986583
*** Phaeoacremonium spadicum ***	**CBS 142711**	**KY906839**	**KY906838**
*** Phaeoacremonium sphinctrophorum ***	**CBS 337.90**	**DQ173113**	**DQ173142**
* Phaeoacremonium sphinctrophorum *	CBS 694.88	DQ173114	DQ173143
*** Phaeoacremonium subulatum ***	**CBS 113584**	**AY579298**	**AY579231**
* Phaeoacremonium subulatum *	CBS 113587	AY579299	AY579232
*** Phaeoacremonium tardicrescens ***	**CBS 110573**	**AY579300**	**AY579233**
*** Phaeoacremonium tectonae ***	**MFLUCC 13-0707**	**KT285563**	**KT285555**
* Phaeoacremonium tectonae *	MFLUCC 14-1131	KT285570	KT285562
*** Phaeoacremonium theobromatis ***	**CBS 111586**	**DQ173106**	**DQ173132**
*** Phaeoacremonium tuscanicum ***	**CBS 123033**	**EU863458**	**EU863490**
*** Phaeoacremonium venezuelense ***	**CBS 651.85**	**AY579320**	**AY579256**
* Phaeoacremonium venezuelense *	CBS 110119	AY579318	AY579254
* Phaeoacremonium venezuelense *	CBS 113595	AY579319	AY579255
* Phaeoacremonium vibratile *	CBS 117115	DQ649063	DQ649064
* Phaeoacremonium viticola *	CBS 113065	DQ173105	DQ173128
* Phaeoacremonium viticola *	CBS 101737	AF246817	DQ173129
*** Pleurostomophora richardsiae ***	CBS 270.33	AY579334	AY579271
* Wuestneia molokaiensis *	CBS 114877	AY579335	AY579272

Abbreviations: **CBS**: CBS-KNAW Collections, Westerdijk Fungal Biodiversity Institute, Utrecht, The Netherlands; **CMM**: Culture Collection of Phytopathogenic fungi “Prof. Maria Menezes”; **CPC**: Culture collection of Pedro Crous, housed at CBS; **HKUCC**: The University of Hong Kong Culture Collection; **ICMP**: The International Collection of Microorganisms from Plants; **KMU**: Kanazawa Medical University herbarium; **MFLU**: Mae Fah Luang University herbarium, **MFLUCC**: Mae Fah Luang University Culture Collection, Chiang Rai, Thailand; **PARC**: Pacific Agri-Food Research Centre.

## Results

### Phylogenetic analyses

**Figure 1.**
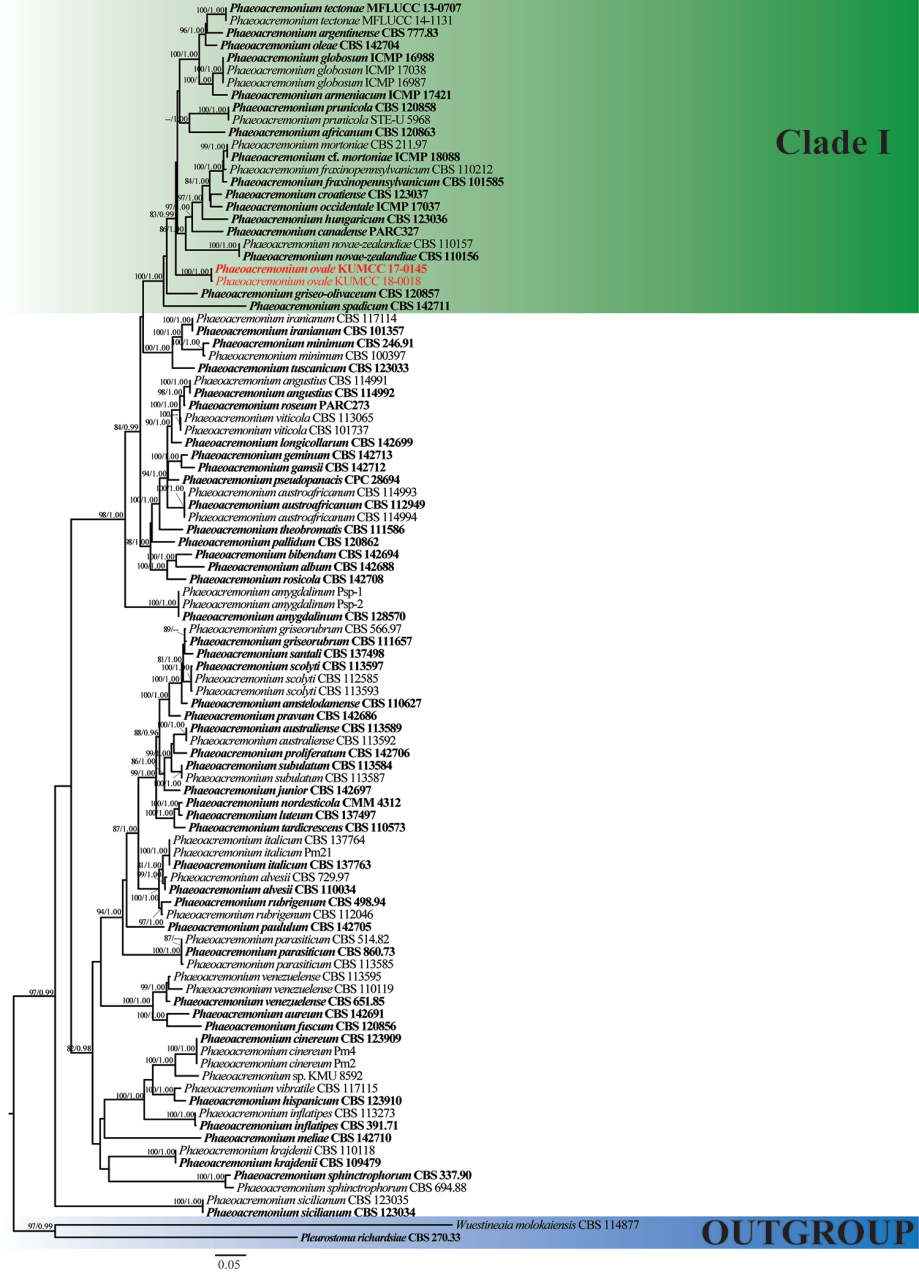
Maximum likelihood phylogenetic tree generated from analysis of a combined TUB and ACT sequences dataset for 98 taxa of Togniniaceae. *Pleurostomarichardsiae* (CBS 270.33) and *Wuestineaiamolokaiensis* (CBS 114877) are the outgroup taxa. ML support values greater than 70% (BSML, left) and Bayesian posterior probabilities greater than 0.90 (BYPP, right) are indicated above the nodes. The strain numbers are noted after the species names. Ex-type strains are indicated in **bold**. Isolates from this study are indicated in red.

The combined TUB and ACT sequence dataset comprised 98 strains of *Phaeoacremonium*. The tree was rooted with *Pleurostomarichardsiae* (CBS 270.33) and *Wuestineaiamolokaiensis* (CBS 114877). The alignment comprised 947 total characters including gaps (TUB: 646bp; ACT: 301bp). ML and BI analyses yielded trees which were topologically congruent in terms of species groupings. RAxML analysis yielded a best scoring tree with a final optimisation likelihood value of -15310.399369 (Fig. 1). In the phylogenetic tree, two strains of *Phaeoacremoniumovale* forms a well-supported independent subclade (100%, ML/1.00, PP) and closely related to other *Phaeoacremonium* species in Clade I (83%, ML/0.99, PP).

### Taxonomy

#### Phaeoacremonium ovale

Taxon classificationFungiTogninialesTogniniaceae

S.K. Huang, R. Jeewon & K.D. Hyde sp. nov.

Fig. 2

##### Type.

CHINA, Yunnan Province, Baoshan, stream along the roadside; saprobic on dead wood, 21 December 2016; Huang S.K. (KUN HKAS99550, holotype; MFLU MFLU18-1076, isotype); ex-type living culture (KUMCC 17-0145; KUMCC 18-0018). GenBank no. (ITS: MH399732, TUB: MH395327, ACT: MH395325; ITS: MH399733, TUB: MH395328, ACT: MH395326)

##### Etymology.

The name *ovale* refers to the oval shaped ascospores.

##### Description.

**Sexual morph**: *Ascomata* 225–300 μm (*n* = 5), on wood, perithecial, solitary, semi-immersed, unilocular, subglobose to globose, black, ostiolate, with ostiolar neck erumpent through bark of host when mature. *Neck* 445–645 × 35–45 μm (*x̄* = 530 × 40 μm, *n* = 5), centrally ostiolate, contorted, lined with hyaline periphyses. *Peridium* 17–40 μm diam., membranous, composed of dark brown to hyaline cells of *textura angularis*. *Hamathecium* composed of 2–6 μm wide, hyaline, septate paraphyses, slightly constricted at septa and gradually narrowed towards apex. *Asci* 11–20 × 3–6 μm (*x̄* = 15.5 × 5 μm, *n* = 30), 8-spored, unitunicate, clavate, with short pedicel, apically rounded. *Ascospores* 3–5 × 1.5–3 μm (*x̄* = 3.5 × 2 μm, *n* = 50), bi-seriate, hyaline, oval to ellipsoid, aseptate, smooth-walled, rounded at the ends. **Asexual morph**: *Mycelium* on culture, partly superficial, composed of septate, branched, hyaline, rarely verrucose, hyphae 1.5–3 μm diam., rarely with adelophialides. *Conidiophores* usually arising from hyaline hyphae, mononematous, unbranched, occasionally constricted at basal septum, hyaline. *Phialides* 8–15 × 2–4 μm (*x̄* = 9.5 × 3 μm, *n* = 20), terminal, monophialidic, elongate-ampulliform and attenuated at base. *Conidia* 2.5–6 × 1–2.5 µm (*x̄* = 4 × 2 μm, *n* = 30), hyaline, ellipsoid to ovoid, aseptate.

**Figure 2.**
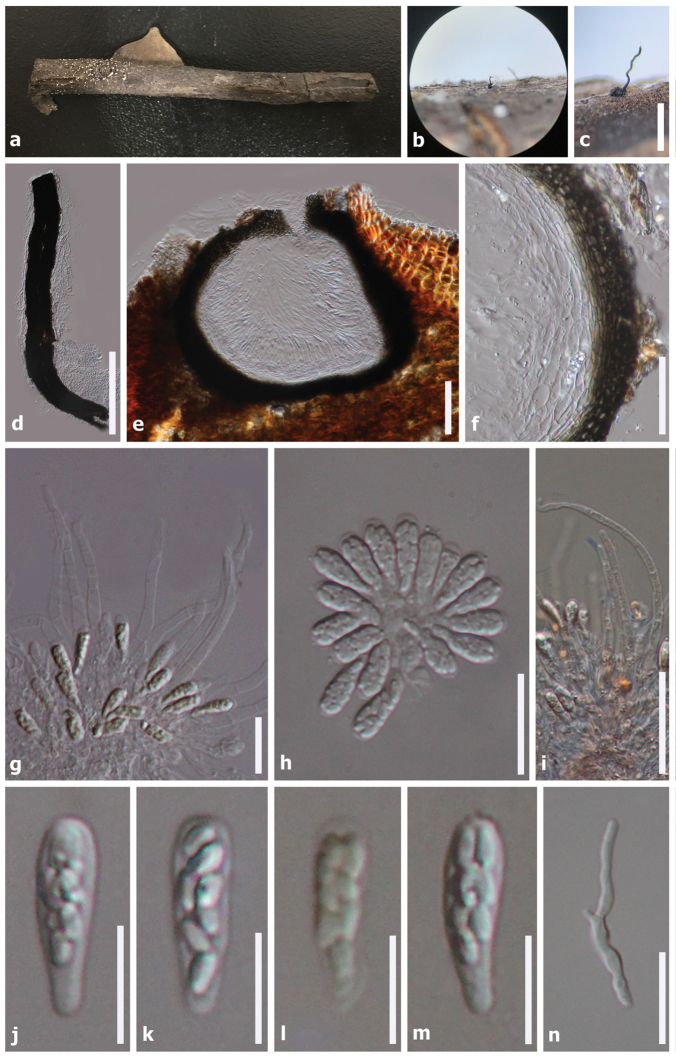
*Phaeoacremoniumovale* (HKAS99550, holotype)**. a** Substrate **b, c** Ascoma on host **d** Squashed neck **e** Ascoma in vertical section **f**Peridium**g** Asci surrounded by paraphyses **h** Asci **i** Septate paraphyses **j–m** Asci with ascospores **n** Germinating ascospores. Note: Fig. i stained in Congo red reagent, fig l stained in Melzer’s reagent. Scale bars: 500 µm (**c**); 200 µm (**d**); 100 µm (**e**); 50 µm (**f, i**); 30 µm (**n**); 20 µm (**g–h**); 10 µm (**j–m**)

##### Culture characteristics.

Ascospore germinating on PDA within 1 week at 23°C, germ tubes produced from ends. Colonies growing on PDA, reaching 2 cm diam. and sporulating after 30 days. Colonies semi-immersed to superficial, irregular in shape, flat, slightly raised, with undulate edge, slightly rough on surface, cottony to fairly fluffy, colony from above, greyish-brown (5F3–5, Ridgway 1912) at the margin, initially write to cream (5A1–3) in the centre, becoming dark brown (5F7–8) at the margin, orange-white (5B1–3) at the centre; from below, initially, greyish-brown at the margin, white at the centre, becoming dark brown at the margin, orange-white at the centre, producing brown pigmentation in agar.

## Discussion

*Phaeoacremonium* is currently accommodated in the monogeneric family Togniniaceae (Wijayawardene et al. 2018). To date, 65 species are accepted in this genus (Mostert et al. 2006b; Gramaje et al. 2015; Marin-Felix et al. 2018; Spies et al. 2018). While most of the species are commonly isolated as asexual morphs, some taxa have been recovered in their sexual morph state, viz. *Phaeoacremoniumaquaticum* (= *Togniniaaquatica*), *P.viticola* (= *T.viticola*), *P.novae-zealandiae* (= *T.novae-zealandiae*) (Hausner et al. 1992; Mostert et al. 2006a; Hu et al. 2012).

**Figure 3.**
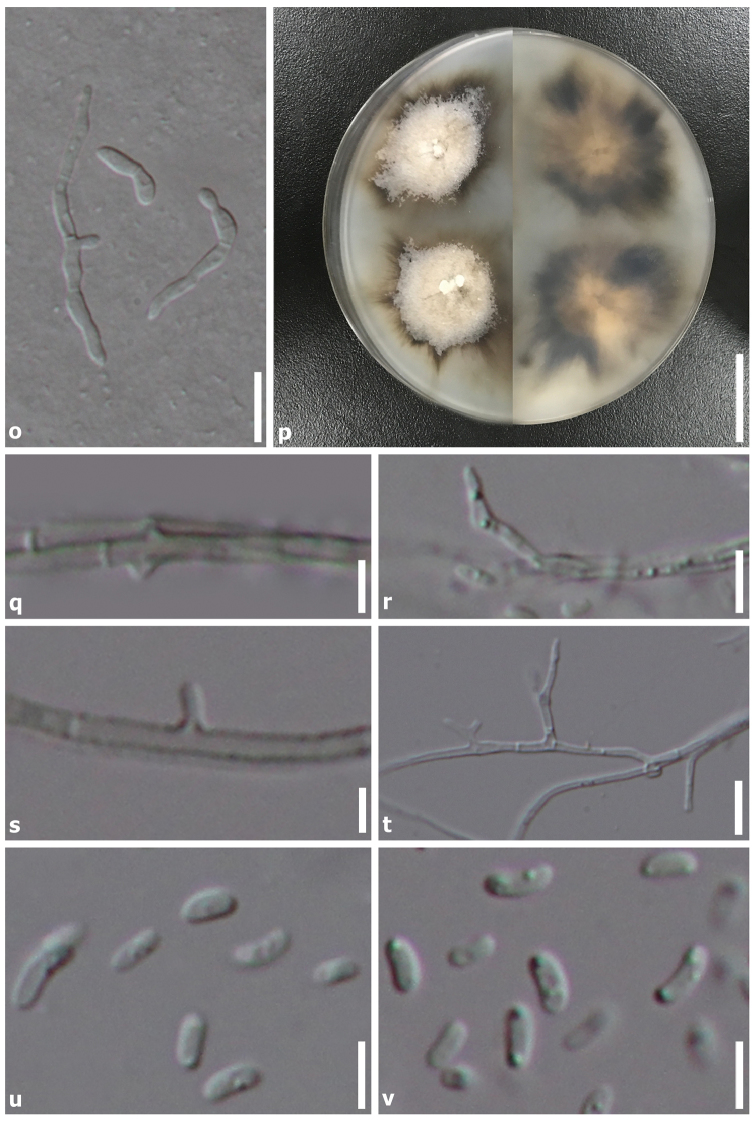
*Phaeoacremoniumovale* (HKAS99550, holotype). **o** Germinating ascospores, **p** 7 weeks of culture plate (above, left/reverse, right), **q** Mycelium with adelophialides **r–t** Branched conidiophores **u–v**Conidia. Scale bars: 20 mm (**p**); 20 µm (**o**); 10 µm (**r, t**); 5 µm (**q, s, u–v**).

In this study, we introduce a novel taxon of *Phaeoacremonium* from dead wood collected in a stream in the Yunnan Province, China and describe its sexual and asexual morph. Examination of morphological characters reveal that our species is sufficiently distinct from extant species to establish it as a new species. Analyses of the combined DNA sequence dataset from partial TUB and ACT genes also support that this taxon is a *Phaeoacremonium* species and phylogenetically distinct from other species (Fig. 1). The two strains of *P.ovale* constitute a strongly supported independent lineage close to other species as depicted in Clade I. Phylogeny also reveals a close relationship to *P.griseo-olivaceum*, but with low support. To further support *P.ovale* as a new species, we compared nucleotide differences with other related species as recommended by Jeewon and Hyde (2016). Comparison of the 533 nucleotides across the TUB region reveals 43 bp (10%) differences, 256 bp of the ACT region reveals 22 bp (8.5%) differences and 517 bp of the ITS region reveals 4 bp (1%) differences compared to *P.griseo-olivaceum* (CBS 120857). Examination of the TUB region reveals 59 bp (11%) difference compared to *P.africanum* (CBS 120863) while the ACT region reveals 19 bp (7%) and ITS region reveals 17 bp (3%) differences, but the latter clusters in a different subclade in our phylogeny and is therefore considered distinct. There are also some morphological similarities between *P.ovale* and *P.africanum* in terms of black ascomata with a long neck, clavate asci and small, oval to ellipsoid ascospores in sexual morph and ellipsoid to ovoid, aseptate conidia in asexual morph (Damm et al. 2008). Despite a morphological resemblance to *P.africanum* and close relationship to *P.griseo*-*olivaceum*, there are other differences across these species. *Phaeoacremoniumovale* was collected from an aquatic habitat and from dead wood in China whereas the former two species were collected from *Prunus* spp. in South Africa (Damm et al. 2008). In addition, conidial size of *P.africanum* and *P.griseo*-*olivaceum* are 5–12 × 1.5–2 µm and 5–8 × 1.5–2 µm, whereas conidia of *P.ovale* measure 2.5–6 × 1–2.5 µm (Damm et al. 2008; Fig. 3). No sequence data of the TUB and ACT gene are available for *P.aquaticum* and *P.leptorrhynchum* and therefore we provide ITS sequences of our strains and compare them with those two species. Comparison of ITS regions reveals 61 bp (12%) differences with *P.aquaticum* (IFRDCC 3035) and 11 bp (2%) differences with *P.leptorrhynchum* (UAMH9590). In addition, our new species is also morphologically different from them. *Phaeoacremoniumovale* is morphologically different as ascospores of *P.aquaticum* and *P.leptorrhynchum* are reniform (ascospores of *P.ovale* are oval/ellipsoid) and measure 5–6 × 1–1.5 µm and 7–10 × 1–1.5 µm, respectively. *Phaeoacremoniuminconspicuum* as described by Gramaje et al. (2015) also appears morphologically similar to *P.ovale* in terms of clavate asci and hyaline, aseptate ascospores (Eriksson and Yue 1990), but could not be included in our analyses as DNA sequences are unavailable. However, the ascospore shape and size of *P.inconspicuum* is different (allantoid, measuring 7–10 × 1.5–2 µm) (Eriksson and Yue 1990; Réblová 2011).

## Supplementary Material

XML Treatment for Phaeoacremonium ovale

## References

[B1] Cabanela MV Jeewon R Hyde KD (2007) Morphotaxonomy and phylogeny of *Paoayensislignicola* gen et sp. nov. (ascomycetes) from submerged wood in Paoay Lake, Ilocos Norte, the Philippines. Cryptogamie, Mycologie 28: 301–310.

[B2] Carbone I Kohn LM (1999) A method for designing primer sets for speciation studies in filamentous ascomycetes. Mycologia 91(3): 553–556. 10.2307/3761358

[B3] Carlucci A Lops F Cibelli F Raimondo ML (2015) *Phaeoacremonium* species associated with olive wilt and decline in southern Italy. European Journal of Plant Pathology 141(4): 717–729. 10.1007/s10658-014-0573-8

[B4] Chomnunti P Hongsanan S Aguirre-Hudson B Tian Q Peršoh D Dhami MK Alias AS Xu JC Liu XZ Stadler M Hyde KD (2014) The sooty moulds. Fungal Diversity 66: 1–36. 10.1007/s13225-014-0278-5

[B5] Crous PW Gams W Wingfield MJ van Wyk PS (1996) *Phaeoacremonium* gen. nov. associated with wilt and decline diseases of woody hosts and human infections. Mycologia 88(5): 786–796. 10.1080/00275514.1996.12026716

[B6] Damm U Mostert L Crous PW Fourie PH (2008) Novel *Phaeoacremonium* species associated with necrotic wood of *Prunus* trees. Persoonia 20: 87–102. 10.3767/003158508X324227 20467488 PMC2865348

[B7] Darriba D Taboada GL Doallo R Posada D (2012) jModelTest 2: more models, new heuristics and parallel computing. Nature Methods 9(8): 772. 10.1038/nmeth.2109 PMC4594756 22847109

[B8] Di Marco S Calzarano F Osti F Mazzullo A (2004) Pathogenicity of fungi associated with a decay of kiwifruit. Australasian Plant Pathology 33: 337–342. 10.1071/AP04024

[B9] Eriksson O Yue JZ (1990) Notes on bambusicolous pyrenomycetes. No.s 1-10. Mycotaxon 38: 201–220.

[B10] Glass NL Donaldson GC (1995) Development of primer sets designed for use with the PCR to amplify conserved gene from filamentous ascomycetes. Applied and Environmental Microbiology 61: 1323–1330. 7747954 10.1128/aem.61.4.1323-1330.1995 PMC167388

[B11] Gramaje D García-Jiménez J Armengol J (2012) Fungal trunk pathogens in Spanish grapevine nurseries: a survey of current nursery management practices in Spain. Phytopathologia Mediterranea 51: 411–412.

[B12] Gramaje D Mostert L Groenewald JZ Crous PW (2015) *Phaeoacremonium*: From esca disease to phaeohyphomycosis. Fungal Biology 119: 759–783. 10.1016/j.funbio.2015.06.004 26321726

[B13] Groenewald M Kang JC Crous PW Gams W (2001) ITS and β-tubulin phylogeny of *Phaeoacremonium* and *Phaeomoniella* species. Mycological Research 105: 651–657. 10.1017/S0953756201004282

[B14] Guarro J Alves SH Gené J Grazziotin NA Muzzuco R Dalmagro C Capilla J Zaror L Mayayo E (2003) Two cases of subcutaneous infection due to *Phaeoacremonium* spp. Journal of Clinical Microbiology 41: 1332–1336. 10.1128/JCM.41.3.1332-1336.2003 12624080 PMC150290

[B15] Hall T (1999) BioEdit: a user-friendly biological sequence alignment editor and analysis program for Windows 95/98/NT. Nucleic Acids Symposium Series 41: 95–98.

[B16] Hausner G Eyjólfsdóttir GG Reid J Klassen GR (1992) Two additional species of the genus *Togninia*. Canadian Journal of Botany 70(4): 724–734. 10.1139/b92-093

[B17] Hemashettar BM Siddaramappa B Munjunathaswamy BS Pangi AS Pattan J Andrade AT Padhye AA Mostert L Summerbell RC (2006) *Phaeoacremoniumkrajdenii*, a cause of white grain eumycetoma. Journal of Clinical Microbiology 44: 4619–4622. 10.1128/JCM.01019-06 17005754 PMC1698411

[B18] Hongsanan S Maharachchikumbura SSN Hyde KD Samarakoon MC Jeewon R Zhao Q Al-Sadi AM Bahkali AH (2017) An updated phylogeny of Sordariomycetes based on phylogenetic and molecular clock evidence. Fungal Diversity 84: 25–41. 10.1007/s13225-017-0384-2

[B19] Hu DM Cai L Hyde KD (2012) Three new ascomycetes from freshwater in China. Mycologia 104(6): 1478–1489. 10.3852/11-430 22684292

[B20] Hyde KD Fryar S Tian Q Bahkali AH Xu JC (2016) Lignicolous freshwater fungi along a north-south latitudinal gradient in the Asian/Australian region; can we predict the impact of global warming on biodiversity and function? Fungal Ecology 19: 190–200. 10.1016/j.funeco.2015.07.002

[B21] Jayasiri SC Hyde KD Ariyawansa HA Bhat J Buyck B Cai L Dai YC Abd-Elsalam KA Ertz D Hidayat I Jeewon R Jones EBG Bahkali AH Karunarathna SC Liu JK Luangsa-ard JJ Lumbsch HT Maharachchikumbura SSN McKenzie EHC Moncalvo JM GhobadNejhad M Nilsson H Pang KL Pereira OL Phillips AJL Raspé O Rollins AW Romero AI Etayo J Selçuk F Stephenson SL Suetrong S Taylor JE Tsui CKM Vizzini A Abdel-Wahab MA Wen TC Boonmee S Dai DQ Daranagama DA Dissanayake AJ Ekanayaka AH Fryar SC Hongsanan S Jayawardena RS Li WJ Perera RH Phookamsak R de Silva NI Thambugala KM Tian Q Wijayawardene NN Zhao RL Zhao Q Kang JC Promputtha I (2015) The Faces of fungi database: fungal names linked with morphology, phylogeny and human impacts. Fungal Diversity 74: 3–18. 10.1007/s13225-015-0351-8

[B22] Jeewon R Cai L Zhang K Hyde KD (2003) *Dyrithiopsislakefuxianensis* gen et sp. nov. from Fuxian Lake, Yunnan, China and notes on the taxonomic confusion surrounding *Dyrithium*. Mycologia 95: 911–920. 10.1080/15572536.2004.11833050 21148998

[B23] Jeewon R Hyde KD (2016) Establishing species boundaries and new taxa among fungi: recommendations to resolve taxonomic ambiguities. Mycosphere 7: 1669–1677. 10.5943/mycosphere/7/11/4

[B24] Katoh K Standley DM (2016) A simple method to control over-alignment in the MAFFT multiple sequence alignment program. Bioinformatics 32(13): 1933–1942. 10.1093/bioinformatics/btw108 27153688 PMC4920119

[B25] Kubátová A Kolarik M Pazoutová S (2004) *Phaeoacremoniumrubrigenum*–hyphomycete associated with bark beetles found in Czechia. Folia Microbiology 49: 99–104. 10.1007/BF02931381 15227778

[B26] Luo ZL Hyde KD Bhat DJ Jeewon R Maharachchikumbura SSN Bao DF Li WL Su XJ Yang XY Su HY (2018) Morphological and molecular taxonomy of novel species *Pleurotheciaceae* from freshwater habitats in Yunnan, China. Mycological Progress 17(5): 511–530. 10.1007/s11557-018-1377-6

[B27] Marin-Felix Y Hernández-Restrepo M Wingfield MJ Akulov A Carnegie AJ Cheewangkoon R Gramaje D Groenewald JZ Guarnaccia V Halleen F Lombard L Luangsaard J Marincowitz S Moslemi A Mostert L Quaedvlieg W Schumacher RK Spies CFJ Thangavel R Taylor PWJ Wilson AM Wingfield BD Wood AR Crous PW (2018) Genera of phytopathogenic fungi: GOPHY 2. Studies in Mycology. 10.1016/j.simyco.2018.04.002 PMC6031069 29997401

[B28] Miller AM Schwartz T Pickett BE He S Klem EB Scheuermann RH Passarotti M Kaufman S O’Leary MA (2015) A RESTful API for access to phylogenetic tools via the CIPRES science gateway. Evol Bioinform 11: 43–48. 10.4137/EBO.S21501 PMC4362911 25861210

[B29] Mostert L Crous PW Groenewald JZE Gams W Summerbell RC (2003) *Togninia* (Calosphaeriales) is confirmed as teleomorph of *Phaeoacremonium* by means of morphology, sexual compatibility and DNA phylogeny. Mycologia 95(4): 646–659. 10.1080/15572536.2004.11833069 21148974

[B30] Mostert L Groenewald JZ Summerbell RC Gams W Crous PW (2006a) Taxonomy and pathology of *Togninia* (Diaporthales) and its *Phaeoacremonium* anamorphs. Studies in Mycology 54: 1–115. 10.3114/sim.54.1.1

[B31] Mostert L Halleen F Fourie P Crous PW (2006b) A review of *Phaeoacremonium* species involved in Petri disease and esca of grapevines. Phytopathologia Mediterranea 45: 12–29.

[B32] Nigro F Boscia D Antelmi I Ippolito A (2013) Fungal species associated with a severe decline of olive in Southern Italy. Journal of Plant Pathology 95(3): 668–668.

[B33] O’Donnell K Cigelnik E (1997) Two divergent intragenomic rDNA ITS2 types within a monophyletic lineage of the fungus *Fusarium* are nonorthologous. Molecular Phylogenetics and Evolution 7: 103–116. 10.1006/mpev.1996.0376 9007025

[B34] Olmo D Gramaje D Agustí-Brisach C Leon M Armengol J (2014) First report of *Phaeoacremoniumvenezuelense* associated with decay of apricot trees in Spain. Plant Disease 98(7): 1001. 10.1094/PDIS-12-13-1198-PDN 30708872

[B35] Pascoe IG Edwards J Cunnington JH Cottral E (2004) Detection of the *Togninia* teleomorph of *Phaeoacremoniumaleophilum* in Australia. Phytopathologia Mediterranea 43: 51–58.

[B36] Réblová M (2011) New insights into the systematics and phylogeny of the genus *Jattaea* and similar fungi of the *Calosphaeriales*. Fungal Diversity 49: 167–198. 10.1007/s13225-011-0099-8

[B37] Réblová M Miller AN Rossman AY Seifert KA Crous PW Hawksworth DL Abdel-Wahab MA Cannon PF Daranagama DA De Beer ZW Huang SK Hyde KD Jayawardena R Jaklitsch W Jones EB Ju YM Judith C Maharachchikumbura SS Pang KL Petrini LE Raja HA Romero AI Shearer C Senanayake IC Voglmayr H Weir BS Wijayawarden NN (2016) Recommendations for competing sexual-asexually typified generic names in Sordariomycetes (except Diaporthales, Hypocreales, and Magnaporthales). IMA Fungus 7(1): 131–153. 10.5598/imafungus.2016.07.01.08 27433444 PMC4941682

[B38] Ridgway R (1912) Color Standards and Color Nomenclature. Washington, DC. 10.5962/bhl.title.144788

[B39] Ronquist F Huelsenbeck JP (2003) Mrbayes 3: Bayesian phylogenetic inference under mixed models. Bioinformatics 19: 1572–1574. 10.1093/bioinformatics/btg180 12912839

[B40] Rooney-Latham S Eskalen A Gubler WD (2004) Ascospore discharge and occurrence of *Togniniaminima* (anamorph = *Phaeoacremoniumaleophilum*) in California vineyards. (Abstr.) Phytopathology 94: S57.

[B41] Rooney-Latham S Escalen A Gubler WD (2005a) Teleomorph formation of *Phaeoacremoniumaleophilum*, cause of esca and grapevine decline in California. Plant Disease 89: 177–184. 10.1094/PD-89-0177 30795221

[B42] Rooney-Latham S Eskalen A Gubler WD (2005b) Ascospore release of *Togniniaminima*, cause of esca and grapevine decline in California. Online. Plant Health Progress. 10.1094/PHP-2005-0209-01-RS 30795221

[B43] Rumbos IC (1986) *Phialophoraparasitica*, causal agent of cherry dieback. Journal of Phytopathology 117: 283–287. 10.1111/j.1439-0434.1986.tb00944.x

[B44] Spies CFJ Moyo P Halleen F Mostert L (2018) *Phaeoacremonium* species diversity on woody hosts in the Western Cape Province of South Africa. Persoonia 40: 26–62. 10.3767/persoonia.2018.40.02 PMC6146639 30504995

[B45] Stamatakis A (2014) RAxML version 8: a tool for phylogenetic analysis and post-analysis of large phylogenies. Bioinformatics 30: 1312–1313. 10.1093/bioinformatics/btu033 24451623 PMC3998144

[B46] Wijayawardene NN Hyde KD Lumbsch HT Liu JK Maharachchikumbura SSN Ekanayaka AH Tian Q Phookamsak R (2018) Outline of Ascomycota: 2017. Fungal Diversity 88: 167–263. 10.1007/s13225-018-0394-8

[B47] White TJ Bruns T Lee S Taylor J (1990) Amplification and direct sequencing of fungal ribosomal RNA genes for phylogenetics. In: Innis MA, Gelfand DH, Sninsky JJ, White TJ. (Eds) PCR Protocols: A Guide to Methods and Applications. Academic Press, San Diego, 315–322.

[B48] Zhang Y Jeewon R Fournier J Hyde KD (2008) Multi-gene phylogeny and morphotaxonomy of *Amniculicolalignicola*: novel freshwater fungus from France and its relationships to the Pleosporales. Fungal Biology 112: 1186–1194. 10.1016/j.mycres.2008.04.004 18783929

